# Understanding recruitment to a randomised controlled trial (RCT) during liver transplantation: an observational mixed-methods Study Within A Trial (SWAT)

**DOI:** 10.1136/bmjopen-2025-104310

**Published:** 2026-01-07

**Authors:** Katherine Elizabeth Sarah Coppack, Ilya Kantsedikas, Edgar Brodkin, Ee Neng Loh, Gareth Ambler, Suneetha Ramani Moonesinghe, Jeremy Fabes, Vivienne Hannon, Michael Spiro, Duncan Wagstaff

**Affiliations:** 1 North Central and East London Rotation, NHS Health Education England London School of Anaesthesia, London, England, UK; 2 North West London Rotation, NHS Health Education England London School of Anaesthesia, London, UK; 3 Anaesthesia, Hillingdon Hospitals NHS Foundation Trust, Uxbridge, UK; 4 Department of Anaesthesia, Royal Free London NHS Foundation Trust, London, UK; 5 Department of Statistical Science and Joint Research Support Office, University College London, London, UK; 6 Centre for Perioperative Medicine, Division of Surgery and Targeted Intervention, University College London, London, UK; 7 Health Services Research Centre, Royal College of Anaesthetists, London, UK; 8 Faculty of Health, University of Plymouth, Plymouth, UK; 9 Department of Anaesthesia, University Hospitals Plymouth NHS Trust, Plymouth, UK; 10 Division of Surgical Biotechnology, University College London, London, England, UK; 11 Intensive Care and Anaesthesia, Royal Free Hospital, London, England, UK; 12 Health Technology Research Centre in Sustainable Innovation, Royal Devon University Healthcare NHS Foundation Trust, Exeter, England, UK

**Keywords:** QUALITATIVE RESEARCH, TRANSPLANT SURGERY, Randomized Controlled Trial, Patient Participation

## Abstract

**Objectives:**

Perioperative randomised controlled trials (RCTs) in liver transplantation are relatively infrequent. RCTs performed in this complex patient population need to be robustly conducted to maximise patient benefit and graft utility given the scarcity of donor organs. Recruitment challenges can compromise RCTs and studies in this population face unique challenges due to recipient illness severity, their comorbidities, demographics and the geographical constraints of specialist transplant centres. Emergency presentation and after-hours admission may further limit patients’ capacity or readiness to consider trial participation. This Study Within a Trial (SWAT) specifically explored motivators and barriers to recruitment in patients awaiting liver transplantation.

**Design:**

An observational mixed-methods ‘Study within a Trial’, nested within a feasibility RCT.

**Setting:**

This study was dual centred at two Tertiary National Health Service Hospitals; The Royal Free Hospital, a liver transplant centre in North London and University Hospital Birmingham, a liver transplant centre in Birmingham.

**Participants:**

Adults who were eligible for liver transplantation and recruitment into the associated RCT were eligible for inclusion into the SWAT.

**Interventions:**

Completion of an 18-question validated written questionnaire which explored motivation for accepting or declining participation in the RCT.

**Main outcome measures:**

Through completion of the questionnaire**,** participants shared their perspectives on the RCT and their rationale for consenting or declining participation. Responses were analysed, providing feedback to the Trial Management Group (TMG) to refine recruitment strategies for future trials. An additional component, comprising interviews and audio recordings of recruitment consultations, was planned if the RCT recruitment rates fell below prespecified thresholds or concerns were raised by the RCT TMG, neither of which occurred.

**Results:**

84 completed questionnaires were received. Motivators included patients believing that the trial will benefit others, interest in helping with research, perception that benefits outweigh risks and belief that it offered the best treatment. Barriers included concerns about randomisation, feeling overburdened and a perception of lack of support from family or friends.

**Conclusion:**

This is the first study exploring recruitment to a perioperative RCT involving patients undergoing liver transplantation. Key motivators were altruism and perceived safety, while barriers included concerns about randomisation and lack of family support. Future focus during recruitment should be on neutral patient-centred consultations, adequate information sharing, fostering of patient trust, improved explanation of randomisation and engagement of the patient’s support network.

**Trial registration number:**

NCT04941911 (Health Research Authority) and SWAT 152 (the Study With A Trial Database).

**STRENGTHS AND LIMITATIONS OF THIS STUDY**

- Qualitative research is particularly important in complex patient groups, such as liver transplant candidates and is relatively uncommon in the transplantation literature.
- The Study Within a Trial used existing infrastructure of the associated feasibility randomised controlled trial.
- The study has provided understanding of personal values and attitudes across two geographically distinct sites.
- The questionnaires being anonymous have limited our ability to explore demographic differences in approaches to recruitment decisions.

## Introduction

 In the UK, end-stage liver disease now represents the fifth most common cause of death in the under 65s with the most rapidly increasing indication for liver transplantation being metabolic dysfunction-associated steatohepatitis.^1^,^3^ Over a 12-month period, between 2022 and 2023, 924 liver transplants were performed in the UK, the second most common solid organ transplantation behind kidney. In March 2023, 663 patients remained active on the liver transplant list, representing an 8% increase from the previous year.^4^

Quantitative research within transplantation medicine has advanced the field considerably and remains crucial to improving care and efficiency.^5^ Robustly conducted randomised controlled trials (RCTs) are the gold standard of quantitative research methodology. They are essential for the effective evaluation of healthcare interventions and increasingly guide the practice and policy of the UK’s National Institute for Health and Care Excellence.^6 7^ RCTs are resource-intensive endeavours with a substantial proportion of the National Institute for Health Research’s annual expenditure of £237.6 million being attributed to their delivery.^7^

A particularly common challenge for RCTs is that of recruitment, and it is estimated that 44% of publicly funded RCTs fail to recruit adequate participant numbers.^7^ Important research questions remain unanswered should RCTs be forced to discontinue prematurely or are inappropriately powered.^8^ The scarcity of donor organs places an imperative to derive the greatest benefit from the available supply, yet questions remain as to the optimal management to achieve this. Robust clinical trials are required to address these questions with adequate recruitment a cornerstone of achieving this. With donor liver grafts already a limited resource, it is even more crucial to strive for robust clinical trials with adequate recruitment levels.^2 9 10^

The UK Medical Research Council recommends the conduct of feasibility/pilot trials to reduce resources being wasted on larger, ultimately non-viable studies if recruitment is considered potentially problematic.^11^ Both quantitative and qualitative strategies are fundamental to the evaluation of feasibility/pilot studies.^11^ A Study within a Trial (SWAT) provides a way to embed qualitative research within a study. They are methodological studies rooted within an ongoing larger clinical trial, feasibility or otherwise. The strength of well-conducted qualitative research in exploring recruitment challenges lies in providing an understanding of patients’ personal values, attitudes and behaviours.^5 11^ A SWAT can be used to evaluate recruitment strategies, explore barriers and facilitators to recruitment and develop approaches as to how to overcome them.^11 12^

Patients awaiting liver transplantation represent a complex group of patients with challenges arising from both patient population and from the nature of the transplantation itself.^2 9 10^ Previous literature has explored potential barriers and enablers to recruitment in non-liver studies, but qualitative studies are relatively uncommon in transplantation literature despite being regarded as even more pertinent in complex patient groups or complex trial environments.^5 8 13^ Benefits have been demonstrated in other fields such as the ProtecT study where tailored feedback during recruitment increased recruitment from 40% to 70% in a multicentred RCT comparing radical surgery, radical conformal radiotherapy and active monitoring in localised prostate cancer.^14 15^

This study is a SWAT nested within the first multicentred RCT to assess the feasibility of randomising patients undergoing liver transplantation to receive either protocolised octreotide infusions or placebo.^16^ With universal and hypothesised liver–patient specific recruitment challenges in mind, and the reported benefit of embedded qualitative research, this ethics approved, dual-centred, cross-sectional SWAT was conducted to explore which factors promote or deter patient recruitment to an associated feasibility RCT.

## Methods

### Study design

This qualitative SWAT (‘the SWAT’) was performed during the recruitment phase of a feasibility RCT (‘the RCT’) of intraoperative octreotide infusion during liver transplantation in two National Health Service hospitals. The study protocol was published prior to commencement of RCT recruitment.^17^

The centres hosting both studies were The Royal Free Hospital (RFH), a liver transplant centre in North London and University Hospital Birmingham (UHB), a liver transplant centre located in Birmingham. RFH served as the lead site for both studies with the teams who led the grant application and study design based there.

The SWAT comprised a written questionnaire which was distributed to all the patients approached for recruitment to the RCT to explore motivation for accepting or declining participation.

An additional component, comprising interviews and audio recordings of recruitment consultations, was planned if the RCT recruitment rates fell below prespecified thresholds or concerns were raised by the RCT Trial Management Group (TMG), neither of which occurred. Prespecified recruitment thresholds for the RCT were set at 0.3 (recruited/approached) at RFH and 0.15 at UHB, based on prior experience with feasibility RCTs in liver transplantation.^18^ These thresholds represented the point at which it was expected that trial recruitment would be incomplete at the end of the recruitment window.

### Patient and public involvement

A patient representative of the TMG who had both liver transplantation and research recruitment experience was actively involved in the review and refinement of both study design and methodology. Study documentation was amended based on their consultation.

### Participants

Recruitment of patients to both the SWAT and RCT occurred in tandem, with the recruitment window spanning 12 months from May 2022 to April 2023. The RFH recruited their first feasibility RCT patient on 27 May 2022, while UHB recruited their first patient on 15 December 2022. All patients eligible for recruitment to the RCT were eligible for inclusion in the SWAT and were asked to complete the SWAT questionnaire irrespective of whether they had consented to recruitment into the RCT. A total of 348 patients were eligible for RCT enrolment and were approached to complete the SWAT questionnaire. The SWAT concluded once recruitment to the RCT ended after achieving the randomisation target of 30 patients.

### Recruitment to the SWAT

Invitation to complete the SWAT questionnaire involved two approaches depending on whether the patient was already on the liver transplant waiting list at the time of the main RCT recruitment window opening or not.

Patients already on the liver transplant waiting list who agreed to be approached for participation in research having been given generic information regarding research were sent the SWAT questionnaire, a patient information sheet (PIS) and a SWAT consent form (for involvement in the interviews if these were triggered) by post along with the RCT study documentation. A telephone recruitment consultation for the RCT took place at least 24 hours following receipt of the documentation. Patients were asked to complete and return the anonymous SWAT questionnaire during this consultation. They were provided with hospital-addressed, prepaid envelopes to return the questionnaires (online supplemental figure 1).

New patients attended a face-to-face preassessment clinic where they were handed the structured questionnaire, a PIS and the SWAT consent form together with the RCT documentation. They were then invited to complete the questionnaire following their RCT recruitment consultation (online supplemental figure 2).

Follow-up phone calls were made by members of the SWAT research team to encourage return of the questionnaires. The return of completed SWAT questionnaires was taken as consent for participation in this SWAT component. Had in-depth interviews been triggered, completion of written consent forms would have been required.

Any personally identifiable information was removed from the returned questionnaires and free text verbatim terms which may have identified patients were generalised prior to publication. The anonymised responses from the returned questionnaires were securely stored and regularly uploaded to an online secure database.

### Data collection

The questionnaire (online supplemental figure 3) drew from a validated instrument used in a previously published study where it was successfully used to explore the motivators and barriers to patients with cancer participating in RCTs.^19^

Through a total of 18 questions, it served to explore a patient’s opinion of clinical trials and the rationale behind their choice to consent to take part or not. 16 questions involved rating factors of potential influence using a 5-point Likert scale, one question required the patient to pick the most important reason for their choice to participate or not and one question allowed a free text answer in which to record any other reasons for their decision.

### Data analysis

A dynamic approach was taken to data analysis of questionnaire responses. Returned questionnaires were analysed throughout the recruitment period and interim findings and themes were fed back to the TMG and recruitment teams of the RCT. The SWAT team feedback included questionnaire response rates, a summary of motivators and barriers to recruitment and differences in those consenting and declining RCT involvement and key free-text themes. These data were analysed with the current and historical RCT recruitment rates to determine whether adjustments were required to trial recruitment or the need to trigger the SWAT interview phase.

On completion of the feasibility RCT, summative analysis was performed on all collected questionnaire data.

A systematic review by Fletcher *et al* previously *abstracted* eight themes (online supplemental figure 4) in relation to recruitment challenges from content analysis of 11 qualitative studies related to recruitment to RCTs.^8^ We adapted this suggested framework and grouped the 16 closed questions into the following themes:

- Effect on patients (perceived harms and benefits).
- Communication (clinician to patient).
- Patient–clinician relationship.
- RCT-specific considerations (understanding of research).

Statistical analysis of the 16 closed questions involved conversion of the Likert response scale to a numeric scale of 1–5, strongly disagree to strongly agree. The respondents were divided and analysed in two groups; those who consented to participate in the RCT (n=75) and those hesitant to participate (declined/undecided, n=7). The median and IQR were described for each question and the groups compared using the two-tailed Mann-Whitney U test. Statistical analysis was performed using GraphPad Prism 10 for macOS, V.10.1.1 (270).

The free-text responses underwent manual thematic analysis by a member of the SWAT team who generated theory-driven initial codes and grouped them informed by the framework described above.

## Results

### Participants

84 patients returned questionnaires. The majority of completed questionnaires were returned by patients who had consented to participate in the RCT (89% n=75, online supplemental Table 1) and this was comparable between sites. The 89% consent rate to RCT participation within the SWAT questionnaire responders compares with an overall recruitment rate of 55% (as prespecified by recruited/approached ratio) to the RCT, and 79% recruitment rate of those patients who had made a recruitment decision by the end of the trial (27% patients had not such a decision).

### Questionnaire completion rates

17% (n=14) of returned questionnaires had at least one unfilled answer and were omitted from the analysis (table 1).

**Table 1:** Percentage completion rate of each closed statement

Question number	Questionnaire statement	*Thematic analysis*	Total completion rate n (%)	Consenting group (n=75)			Hesitant group (n=7)			P value
				Completion rate n (%)	Median response (Likert scale 1–5)	IQR	Completion rate n (%)	Median response (Likert scale 1–5)	IQR	
1	I thought the trial/study offered the best treatment available.	*Effect on patients*	81 (99)	74 (97)	4.0	3.0–5.0	7 (100)	4.0	2.0–5.0	0.69
2	I believed the benefits of treatment in the trial/study would outweigh any side effects.	*Effect on patients*	82 (100)	75 (100)	4.0	4.0–5.0	7 (100)	4.0	3.0–4.0	0.06
3	I was satisfied that either treatment in the trial/study would be suitable for me.	*Effect on patients*	81 (99)	74 (97)	4.5	4.0–5.0	7 (100)	3.0	3.0–4.0	**<0.005**
4	I was worried that my illness would get worse unless I joined the trial/study.	*Effect on patients*	81 (99)	74 (97)	1.0	1.0–4.0	7 (100)	3.0	1.0–4.0	**<0.05**
5	The idea of randomisation worried me.	*RCT-specific considerations*	81 (99)	74 (97)	1.0	1.0–2.0	7 (100)	3.0	3.0–5.0	**<0.0005**
6	I wanted the doctor to choose my treatment rather than be randomised by computer.	*RCT-specific considerations*	81 (99)	74 (97)	2.0	1.0–4.0	7 (100)	5.0	3.0–5.0	**<0.005**
7	The doctor told me what I needed to know about the trial.	*Communication*	81 (99)	74 (97)	5.0	4.0–5.0	7 (100)	4.0	3.0–5.0	**<0.05**
8	I trusted the doctor treating me.	*Patient–clinician relationship*	82 (100)	75 (100)	5.0	5.0–5.0	7 (100)	5.0	4.0–5.0	0.36
9	I was given too much information to read about the trial.	*Communication*	78 (95)	71 (95)	2.0	1.0–3.0	7 (100)	3.0	1.0–4.0	0.14
10	I was given enough information to read about the trial.	*Communication*	78 (95)	71 (95)	5.0	4.0–5.0	7 (100)	4.0	3.0–5.0	0.29
11	I knew that I could leave the trial at any time and still be treated.	*Communication*	78 (95)	71 (95)	5.0	5.0–5.0	7 (100)	5.0	4.0–5.0	0.16
12	I did not feel able to say no.	*Patient–clinician relationship*	77 (94)	71 (95)	1.0	1.0–1.0	6 (86)	2.0	1.0–3.3	0.07
13	I wanted to help with the doctors’ research.	*Patient–clinician relationship*	79 (96)	72 (96)	5.0	5.0–5.0	7 (100)	5.0	3.0–5.0	**<0.05**
14	I feel that others will benefit from the results of the trial.	*Effect on patients*	78 (95)	71 (95)	5.0	5.0–5.0	6 (86)	4.5	2.8–5.0	**<0.005**
15	The doctor wanted me to join the trial.	*Patient–clinician relationship*	78 (95)	71 (95)	3.0	1.0–4.0	7 (100)	3.0	2.0–4.0	0.74
16	Others (eg, family/friends) wanted me to join the trial.	*Effect on patients*	77 (94)	70 (93)	3.0	1.0–4.0	7 (100)	3.0	1.0–4.0	0.87
17	Which is the most important reason for you out of this list?		72 (89)	66 (88)	n/a	n/a	6 (86)	n/a	n/a	n/a
**Average completion rate:**			97%	96%*			98%			

Median and IQR values for Likert scale responses 1–5, from strongly disagree to strongly agree to each question from consent and hesitant groups and p values calculated using the two-tailed Mann-Whitney U test.

Statistically significant p-values are shown in bold

* Three of the questionnaires returned from those who had consented did not have the overleaf section completed, comprising questions 9–18.

n/a, not applicable; RCT, randomised controlled trial.

### Results from closed questions

#### Effect on patients

**Figure 1:**
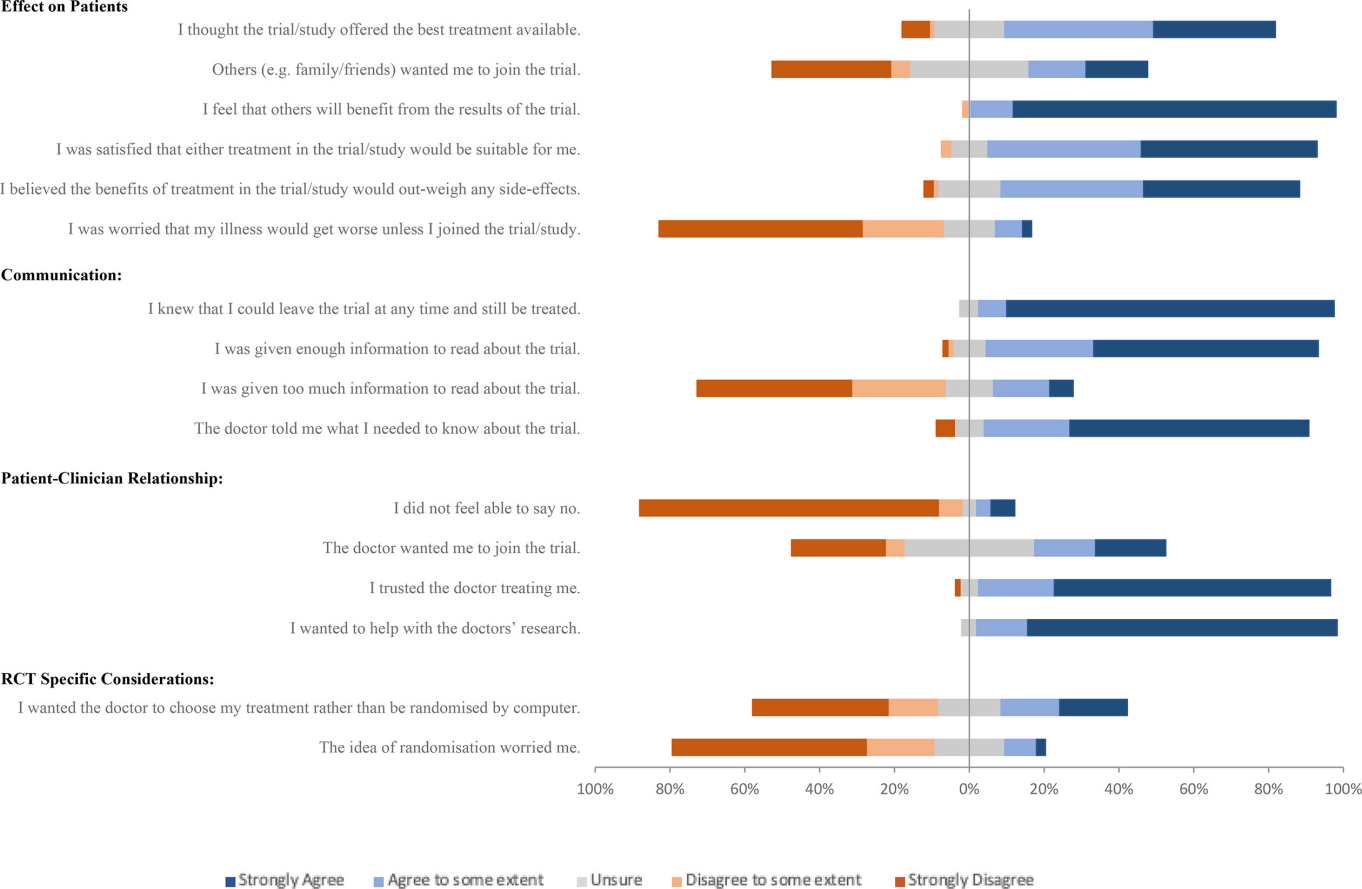
All survey responses to closed questions. Questions divided thematically. Responses are ungrouped. Bars to the right of 0% represent positive responses; agree and strongly agree. Bars to the left of the 0% represent negative responses; disagree and strongly disagree. Unsure responses are divided equally around 0%. Each full bar represents 100% of responses.

Participants agreed that ‘others will benefit from the trial” (table 1, figures1  2). This was also the most common response given (by 50.7%) when participants were asked which was the most important reason for their decision (online supplemental table 2 and figure 5). Participants also agreed that either treatment in the study was suitable, that benefits outweighed risks and that the study offered the best treatment. They disagreed that they were worried that their illness would get worse if they did not participate, and they did not think others wanted them to join the trial.

**Figure 2:**
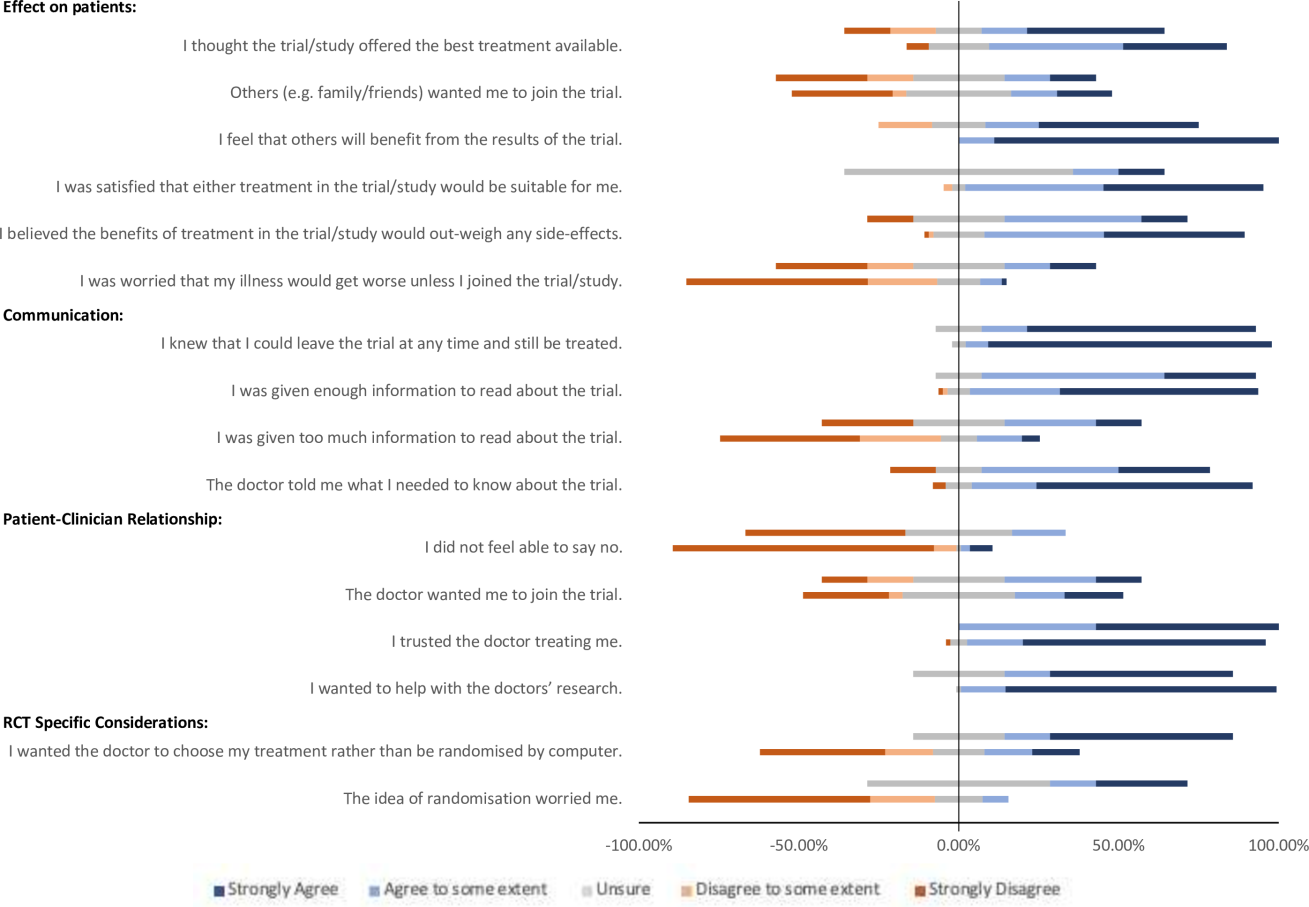
All survey responses to closed questions. Questions divided thematically. Responses are grouped into ‘consenting’ (C) and ‘hesitant’ (H) groups. Bars to the right of 0% represent positive responses; agree and strongly agree. Bars to the left of the 0% represent negative responses; disagree and strongly disagree. Unsure responses are divided equally around 0%. Each full bar represents 100% of responses.

**Table 2:** Thematic analysis of motivations and barriers to participation in the associated feasibility RCT

Example patient quotes	Initial codes	Framework themes
‘Without ongoing research there will be no progress and no new treatments’. ‘My previous background as a healthcare* worker leans me towards research and analysis. Evidence based practice provides confidence in patients needing treatment and clinicians providing the treatment’.	Advancing science	RCT-specific considerations
	Personal connections to research	
	Perceived equivalence of treatment	
	Contribution to the confidence of patients in treatment options	
‘The trial/research would benefit other liver patients without jeopardising my care’. ‘I am waiting for my 3 children to be tested for polycystic liver and kidney disease and feel strongly that any research may help them in the future’.	Benefit to strangers	Effects on patients
	Helping family members	
	Preventing the suffering of others	
‘I only have one kidney, so treatment after transplant needs to protect that remaining’. ‘I feel that I would have too much else going on what with the transplant and having a repair being conducted on a hernia. Also, this is the second study I have been sent to participate in’.	Helping self	Effects on patients
	Desire for new treatments	
	Medical overburden	
	Competing research	
	Perceived responsibility	
‘I have a genuine interest in developing treatments that could potentially help other patients avoid going through what I have gone through. I have been given a lot of support by the medical team and if I can assist in any way it makes me feel like I am providing something in return’.	Giving back to healthcare providers	Patient–clinician relationship
	Contribution to the confidence of healthcare professionals in treatments	
‘It appears that the likelihood of me receiving the drug is about the same whether I participate or not’.	Safety	Effects on patients
	Absence of risk to self	

* Response generalised from specific role for anonymity.

RCT, randomised controlled trial.

There were statistically significant differences between the ‘consenting’ (C) and ‘hesitant’ (H) groups in three questions within this theme (table 1). The consenting group agreed more strongly ‘that others will benefit from the results of the trial’ (C:H median (IQR) 5.0 (5.0–5.0) vs 4.5 (2.8–5.0) p<0.005).

The consenting group also more strongly agreed that ‘either treatment in the trial/study would be suitable for them’ (C:H median (IQR) 4.5 (4.0–5.0) vs 3.0 (3.0–4.0) p<0.005).

The consenting group disagreed more strongly that they were ‘worried about their illness worsening unless they joined the trial’ (C:H median (IQR) 1.0 (1.0–4.0) vs 3.0 (1.0–4.0) p<0.05).

#### Communication

87% of patients agreed that the doctor told them what they needed to know about the trial, with the majority (64%) strongly agreeing. The consenting group agreed more strongly with this statement (C:H median IQR 4.0 (4.0–5.0) vs 4.0 (3.0–5.0) p<0.05) (table 1, figures1  2).

Most patients were satisfied with the amount of information received regarding the trial; 66% disagreed that they had received too much information, while 89% agreed that they had been given enough. 86% of patients agreed that they were aware that they could leave the trial at any time.

#### Patient–clinician relationship

94% of participants reported trusting the doctors treating them. 86% disagreed that they did not feel able to decline participation. Patients were even uncertain as to whether they thought their doctor wanted them to take part.

Consenting participants agreed more strongly that they ‘wanted to help with the doctor’s research’ (C:H median (IQR) 5.0 (5.0–5.0) vs 5.0 (3.0–5.0) p<0.05, Table 1) and 17.8% of respondents chose this statement when prioritising their decision to participate (online supplemental table 2 and figure 5). This was the second most common response and occurred only in the group who had consented to participation (online supplemental table 2 and figure 5).

#### RCT specific considerations

Consenting patients were more likely to disagree with the statement ‘the idea of randomisation worried me’ than those in the hesitant group (C:H median (IQR) 1.0 (1.0–2.0) vs 3.0 (3.0–5.0) p<0.0005) (figure 2, table 1).

Hesitant patients were more likely to agree that they ‘wanted the doctor to choose my treatment rather than be randomised by computer’ (C:H median (IQR) 2.0 (1.0–4.0) vs 5.0 (3.0–5.0) p<0.005). 16.7% of respondents from the hesitant group chose ‘I wanted the doctor to choose my treatment rather than be randomised by computer’ when asked which single statement is the most important factor for their decision (online supplemental table 2 and figure 5)

### Thematic analysis of open question; ‘are there any other reasons for your decision?’

Only n=8 (9.5%) of questionnaire respondents answered the open question. Thematic analysis illustrated several initial codes and mapped them to the analytical framework, as demonstrated in table 2. Six of the comments were interpreted as motivators, one as a barrier and one as a neutral comment (online supplemental figure 6). Five comments related to effects on patients, with motivators being benefits to their own medical condition, their children’s future medical well-being, benefit for other similar patients and reassurance about the safety of the trial. One patient described being overburdened with other medical interventions and research studies. Two patients emphasised the importance of research, and one described trying to ‘provide something in return’ to their medical team.

## Discussion

This is the first study assessing the enablers and barriers of patients undergoing consent for a RCT in liver transplantation. Motivators for participation included hopes that others would benefit, a desire to help doctors with their research and the belief that either treatment seemed suitable. Barriers to recruitment included hesitation surrounding the concept of randomisation, being medically overburdened and the perception that family and friends did not want them to participate.

The most common reason for agreement to participation identified was that ‘others would benefit from the results of the trial’. Altruism as a central motivating theme is noted in existing literature,^20 21^ and our study demonstrates that this is a key enabler in our population. Patients with end-stage liver disease awaiting liver transplant represent a complex cohort of patients both medically and psychologically. Their chronic illness is associated with unpredictable symptom onset and severity, concern regarding deterioration and uncertainty over the future. The wait for a transplant represents a time during which they can experience deterioration in their physical health, including fatigue and weakness, alongside a reduction in their cognitive ability and memory through development of encephalopathy. Waiting on the transplant list is a time of significant uncertainty and stress burden and can contribute to depressive symptoms and negative quality of life. These experiences may provide an additional incentive to consent to research.^19^

The second most frequent motivator was the desire to ‘help the doctors with their research’. This statement is similarly rooted in altruism, but it also highlights the influence of healthcare providers and emphasises the importance of the patient–clinician relationship. Patients awaiting transplantation have been described as having ‘illness uncertainty’, which refers to challenges associated with chronic illness whereby they can have difficulty in understanding treatments or healthcare systems and can experience inconsistent messages from healthcare providers.^22^ Our patient group reported that doctors told them what they needed to know about the trial. Patients awaiting liver transplantation represent a population who have frequent engagement with healthcare professionals over a potentially long time period and may well have developed relationships and healthcare beliefs. Our patients reported that they received adequate information, trusted their doctors, wanted to give back to healthcare providers and contribute to the confidence of clinicians in treatments. These have been documented as potential barriers in existing literature which were not found in our population and perhaps the chronicity of their illness and prior engagement with a multitude of healthcare professionals goes some way to explain their trust in clinicians and their motivation to help with research.^8^

Interestingly, the hesitant group chose ‘I trusted the doctor treating me’ as their most frequent reason for their decision. Clinician influence and their attitude towards the trial are important considerations in the recruitment process.^23 24^ It has been found that clinicians involved in RCTs can feel conflicted in their role, aware of a mistrust of patients in researchers. Clinicians are tasked with offering trial involvement to all eligible patients but can feel a subsequent responsibility for those enrolled, or concern over a perceived loss of trust from those patients who decline.^8 23 24^ Perhaps the hesitant patient group interpreted during their consultation that the clinician was not encouraging enrolment and trusted this advice.

There was an equal split as to whether patients thought that their doctors wanted them to join the trial. Together with the finding that patients felt that they could decline participation, this suggests that our clinicians found the right balance of supporting patients through a potentially difficult decision without coercion or pressure.^24^

The benefits of participation outweighing risks were the third most endorsed statement and patients felt that either treatment was suitable. The literature describes general recruitment reluctance due to apprehension over potential side-effects, feelings of uncertainty, being uncomfortable with experimentation, trials not being appropriate for serious disease and quality of life being reduced.^23 24^ Transplantation medicine is already unpredictable, patients already feel uncertain and are living with significant symptom burden.^20^ In addition, the transplantation process is a major surgical procedure requiring scheduled postoperative intensive care. Conceivably, on the background of such a significant medical intervention, the administration of octreotide or placebo may feel insignificant in comparison.

The randomisation process of RCTs is frequently quoted in the literature as a barrier to recruitment.^823^,^25^ Although overall our population was not worried about randomisation and did not show a preference over doctor choice versus computer randomisation, the hesitant group demonstrated a greater uncertainty towards the process and further to this, they were more likely to prefer doctor-chosen treatment over randomisation. Waiting for a liver transplant is a period of extreme uncertainty with escalating worry surrounding the availability of a donor organ, the patient’s physical health and the perceived burden on their family.^21^ Perhaps there is a desire within our population to avoid the concept of further ‘chance’, which may be their perception of randomisation. Relinquishing decision making and accepting that the treatment choice will be uncertain can be challenging to rationalise.^8 23 24^

The study has demonstrated that most of the patients did not think their friends or family wanted them to take part. Organ transplantation is complex and dynamic, and caregivers are integral to the process, providing support both within and outside the formal hospital setting. Patients with chronic disease can feel burdensome to their support network and potentially are reluctant to increase that perceived load.^21 26^

RCT participation can be demanding, and one patient in our study explicitly reported that they ‘had too much going on’ and hence declined participation. Patients with end-stage liver disease often face a substantial treatment burden, including frequent outpatient appointments, investigations and complex medication regimens, all of which can contribute to logistical and psychological overload when combined with trial participation.^2^ Recruitment is further complicated by the emergency or unplanned nature of some admissions, as well as out-of-hours presentations, which may reduce opportunities for timely and appropriate engagement with potential participants.^27^ Liver transplant candidates represent a population who can be approached by multiple research teams, and indeed some of our participants were, potentially contributing to participant fatigue or reluctance to engage. The demands of the study should be minimised as much as practicable to reduce the risk of overburden. It is for this reason that we did not initiate in-depth qualitative interviews as part of our recruitment assessment strategy.

A suggestion as to why recruitment was satisfactory compared with existing trial literature may be the patient perception that the feasibility RCT was less experimental than other RCTs. Despite clinical equipoise, Octreotide is already being used in some liver transplant centres.^16^ One patient reported, ‘It appears that the likelihood of me receiving the drug is about the same whether I participate or not’. If risk perception is less, this may have contributed to successful recruitment.

### Limitations

Patients already on the liver transplant waiting list had their information packs posted to them, and we experienced challenges with our post not arriving and questionnaires having to be re-sent. The questionnaire was sent alongside the PIS and consent form for the interviews, which may have contributed to patients feeling overburdened. Furthermore, not all domains of the returned questionnaires were completed. Non-response to questionnaires has been found to be significantly higher in long questionnaires rather than short.^28^ Our questionnaire spanned two A4 sides and perhaps the use of fewer questions in future questionnaires exploring recruitment could be considered.

The decision to keep questionnaires anonymised was a deliberate attempt to avoid concerns regarding confidentiality and lower the threshold for participation. However, by not collecting demographic data such as age, gender or diagnosis, we are restricted in our ability to contextualise responses or assess variability in barriers and enablers across patient subgroups.

The majority of completed questionnaires were returned by patients who consented to participate in the RCT, introducing potential response bias and limiting our understanding particularly of the barriers to recruitment. As a result, the perspectives of those who declined or were undecided about trial participation are likely under-represented. Non-response to the questionnaire may reflect similar reasons for declining RCT trial participation, such as feeling overburdened or experiencing research fatigue.^2 24^ Practical factors may also have contributed to non-response: the SWAT and RCT paperwork were distributed concurrently, which may have exacerbated the perceived administrative burden of participation or led some patients to mistakenly perceive that the questionnaires were directly tied to trial enrolment.

The small number of free-text responses (n=8) represents a clear limitation for thematic analysis, restricting both the depth and diversity of qualitative insights. Although the responses offer valuable illustrative views, the findings should be interpreted with caution and considered exploratory rather than representative. While the study design included a potential interview phase to supplement qualitative exploration, this was not initiated, as recruitment rates remained above prespecified thresholds. The absence of in-depth interviews constrained our ability to more fully explore recruitment dynamics, as interviews allow for richer and more nuanced understanding.^29^ Nevertheless, a commonly cited barrier to recruitment, both in the literature and echoed in our study, is the perception of ‘high burden’.^24^ We specifically designed the study to minimise patient burden by avoiding additional interviews during a particularly sensitive time.

## Conclusion

This SWAT has demonstrated the utility of embedding qualitative research within an RCT of patients awaiting liver transplantation to investigate recruitment in this complex population. We have demonstrated key motivators influencing our population’s decisions and provided timely feedback to the RCT team. Patients awaiting liver transplantation were willing to participate in the RCT with their main motivations being altruistic. Concern over randomisation and perception that family and friends do not endorse their participation were identified as potential barriers. These findings build on the challenges of recruitment which have been previously identified in systematic reviews involving other illnesses.

Recommendations for future RCTs in this population include that focus should continue to be on neutral patient-centred recruitment consultations with adequate information sharing and fostering of patient trust. Attention could be directed towards improving explanation of randomisation and engagement of the patient’s support network.

## Supplementary material

10.1136/bmjopen-2025-104310 online supplemental file 1

## Data Availability

Data are available upon reasonable request.
