# Supplementary material for: Understanding recruitment to a randomised controlled trial (RCT) during liver transplantation: an observational mixed-methods Study Within A Trial (SWAT)
Source: BMJ Open. 2026 Jan 7;16(1):e104310. doi: 10.1136/bmjopen-2025-104310 (PMC12781991; doi:10.1136/bmjopen-2025-104310)
Supplement: online supplemental file 1 [file bmjopen-16-1-s001.pdf]

Appendices:

1.

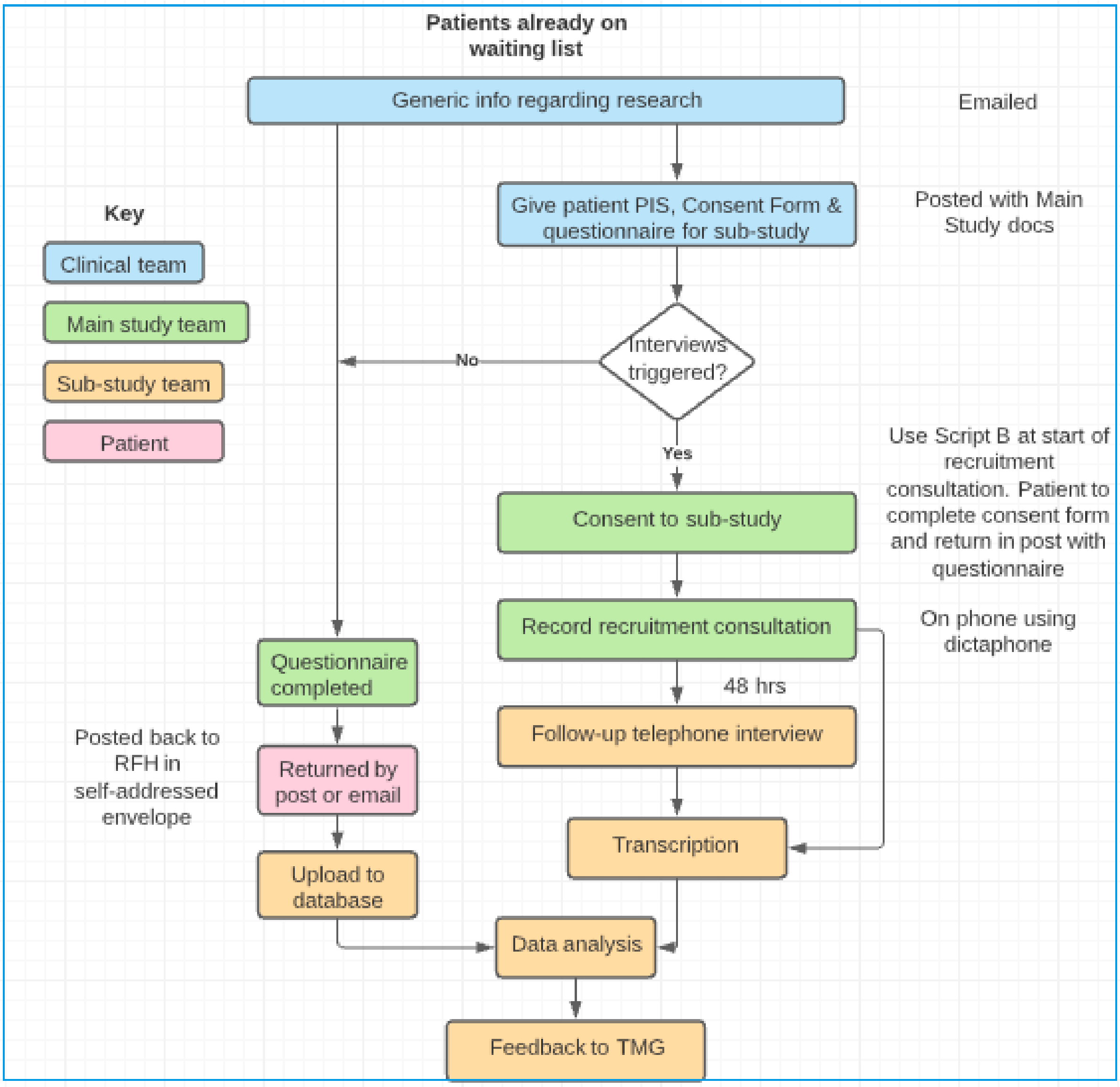

Figure 1: Flowchart demonstrating recruitment to the SWAT for patients on the liver transplant waiting list at time of the RCT recruitment window opening.

2.

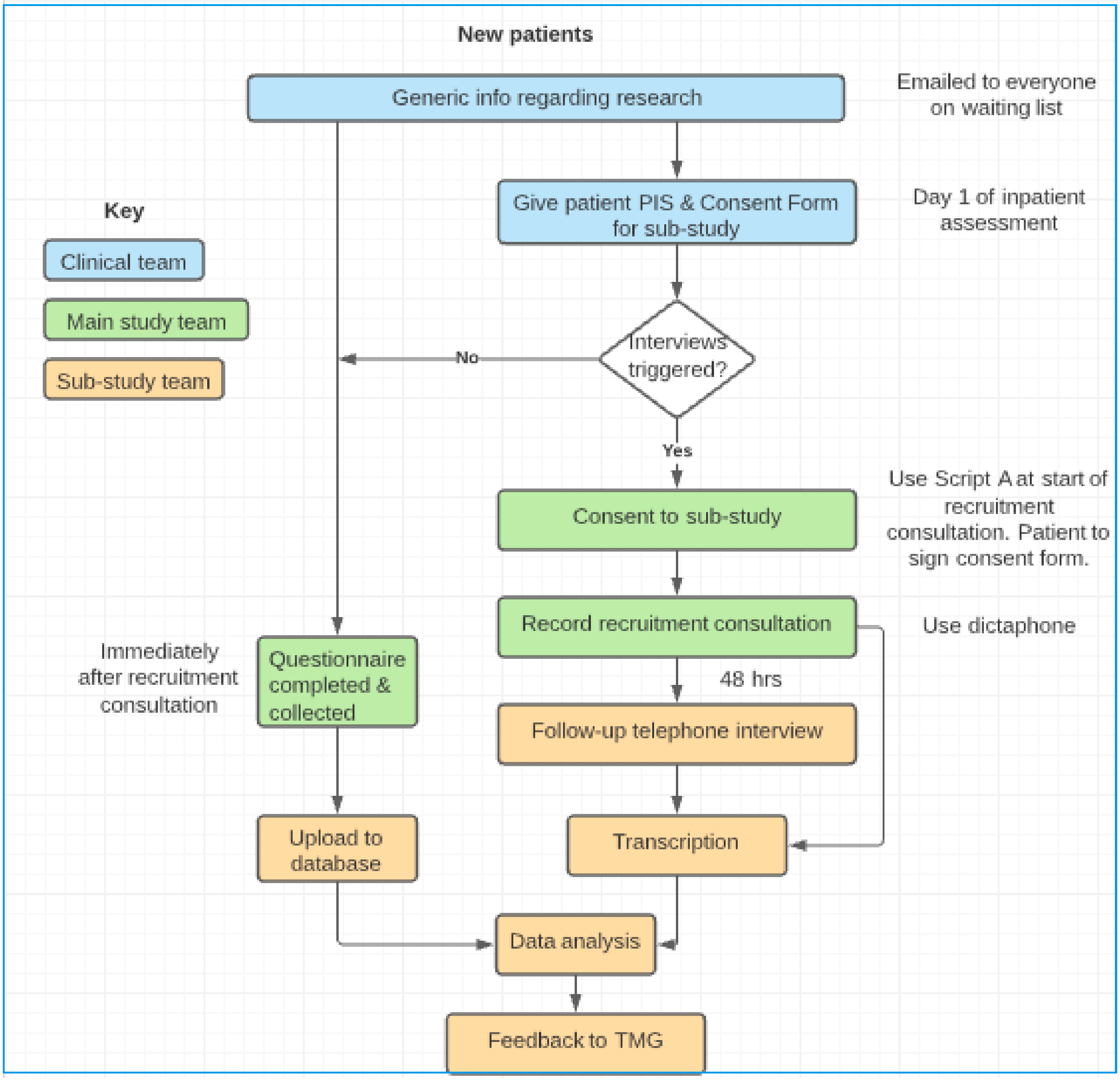

Figure 2: Flowchart demonstrating recruitment to the SWAT for new patients within trial window.

3:

Figure 3: Validated Questionnaire sent to eligible patients. Adapted from: Jenkins V, Fallowfield L. Reasons for accepting or declining to participate in randomized clinical trials for cancer therapy. Br J Cancer. 2000 May 5;82(11):1783–8.

Clinical Trials Questionnaire

We are interested in the reasons why patients accept or decline to take park in the clinical trial of octreotide infusion during liver transplantation. We would be grateful if you could complete and this questionnaire. It will not be shown to your doctor or any of the staff at the hospital.

	Yes	No	Not decided yet
Did you agree to take part in the trial mentioned above?			

Below are some reasons that may have influenced your decision to accept or decline to take part in this trial. Please answer each question by ticking the box that shows most clearly how you feel.

	Strongly agree	Agree to some extent	Unsure	Disagree to some extent	Strongly disagree
1) I thought the trial/study offered the best treatment available.					
2) I believed the benefits of treatment in the trial/study would out-weigh any side-effects.					
3) I was satisfied that either treatment in the trial/study would be suitable for me.					
4) I was worried that my illness would get worse unless I joined the trial/study.					
5) The idea of randomisation worried me.					

6) I wanted the doctor to choose my treatment rather than be randomised by computer.					
7) The doctor told me what I needed to know about the trial.					
8) I trusted the doctor treating me.					
9) I was given too much information to read about the trial.					
10) I was given enough information to read about the trial.					
11) I knew that I could leave the trial at any time and still be treated.					
12) I did not feel able to say no.					
13) I wanted to help with the doctors' research					
14) I feel that others will benefit from the results of the trial.					
15) The doctor wanted me to join the trial.					
16) Others (e.g. family/friends) wanted me to join the trial.					

Which as the most important reason for you out of this list? (Please give number)_______________

Any other comments:

4.

Figure 4: Abstracted themes from Fletcher *et al* regarding recruitment challenges [8]

- Understanding of research (in general; RCTs; in- light of specific trials)
- Communication (clinician to patient; clinician to trial coordinator)
- Perceived patient barriers
- Patient clinician relationship
- Effect on patients (harms and benefits)
- Effects on clinical practise
- Individual benefits for clinicians
- Methods associated with successful recruitment

	Royal Free Hospital (n = 50)		University Hospital Birmingham (n = 34)		Total (n = 84)	
Consented	86%	(n=43)	94%	(n=32)	89%	(n=75)
Declined	4%	(n=2)	6%	(n=2)	5%	(n=4)
Not yet decided	6%	(n=3)	0%		4%	(n=3)
Unclear	4%	(n=2)	0%		2%	(n=2)

5.

Table 1: Main RCT recruitment decisions for patients returning SWAT questionnaires.

6.

	% Response		
**Survey Statement**	**Total n = 73 (%)**	**Consented group n = 67 (%)**	**Hesitant group n = 6 (%)**
I feel that others will benefit from the results of the trial.	37 (50.7)	36 (53.7)	1 (16.7)
I wanted to help with the doctors' research.	13(17.8)	13 (19.4)	0 (0)
I believed the benefits of treatment in the trial/study would out-weigh any side-effects.	8 (11.0)	7 (10.5)	1 (16.7)
I thought the trial/study offered the best treatment available	6 (8.2)	5 (7.5)	1 (16.7)
I was satisfied that either treatment in the trial/study would be suitable for me.	5 (6.8)	5 (7.5)	0 (0)
I wanted the doctor to choose my treatment rather than be randomised by computer	2 (2.7)	1 (1.5)	1 (16.7)
I trusted the doctor treating me	2 (2.7)	0 (0)	2 (33.3)
I was worried that my illness would get worse unless I joined the trial/study.	0 (0)	0 (0)	0 (0)
The idea of randomisation worried me.	0 (0)	0 (0)	0 (0)
The doctor told me what I needed to know about the trial	0 (0)	0 (0)	0 (0)
I was given too much information to read about the trial.	0 (0)	0 (0)	0 (0)
I was given enough information to read about the trial.	0 (0)	0 (0)	0 (0)
I knew that I could leave the trial at any time and still be treated.	0 (0)	0 (0)	0 (0)
I did not feel able to say no.	0 (0)	0 (0)	0 (0)
The doctor wanted me to join the trial.	0 (0)	0 (0)	0 (0)
Others (e.g. family/friends) wanted me to join the trial.	0 (0)	0 (0)	0 (0)

Table 2: Response to Question 17**;** "Which is the most important reason for you out of this list?" in terms of a decision to consent or not to participation in the RCT

7:

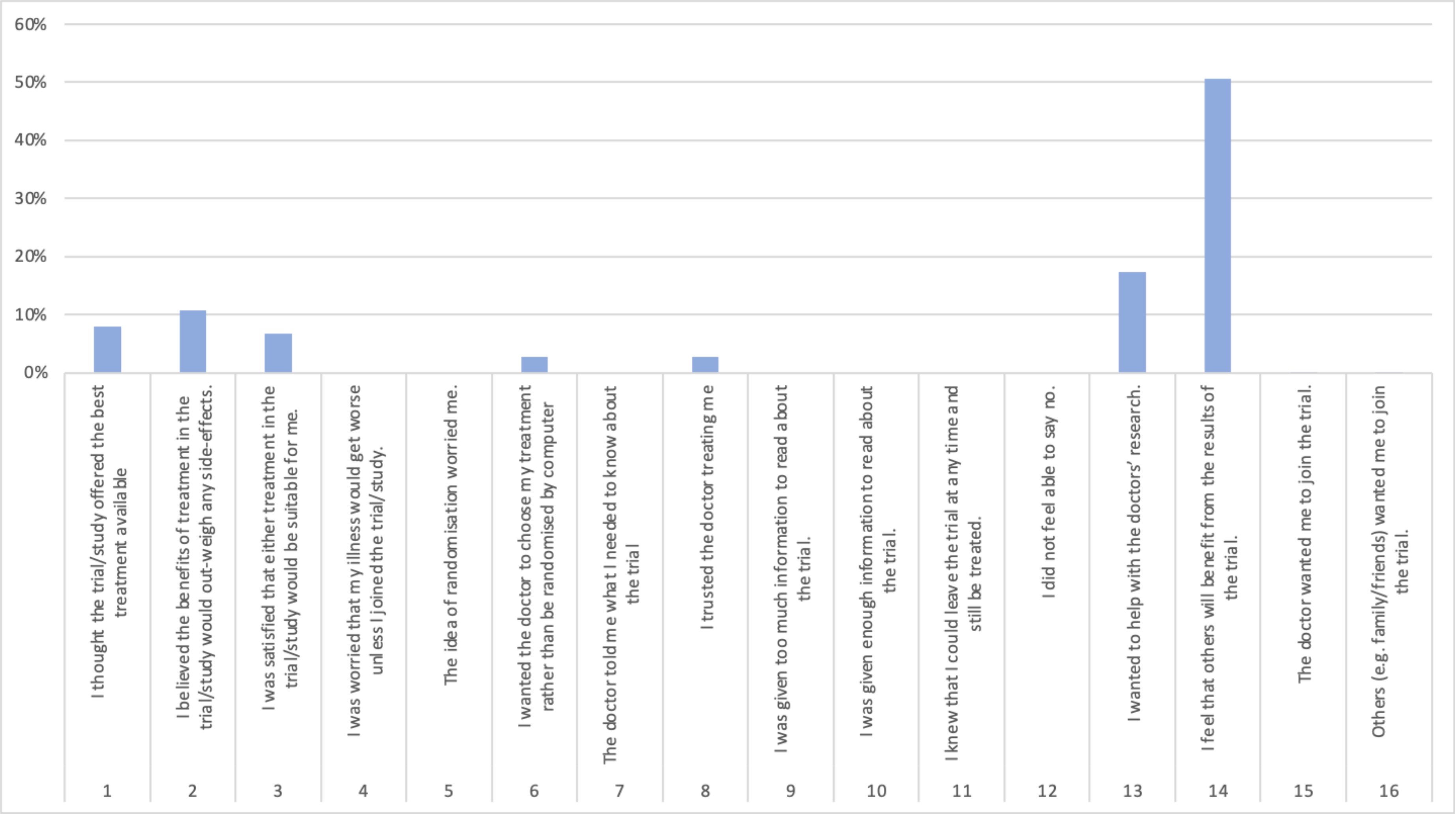

Figure 5: Response to the question "Which is the most important reason for you out of this list?" in terms of a decision to consent or not to participation in the RCT

8:

Thematic analysis of open question; “Are there any other reasons for your decision?”

9.5% of questionnaire respondents completed the open question field (n=8). The eight responses are demonstrated in figure 6.

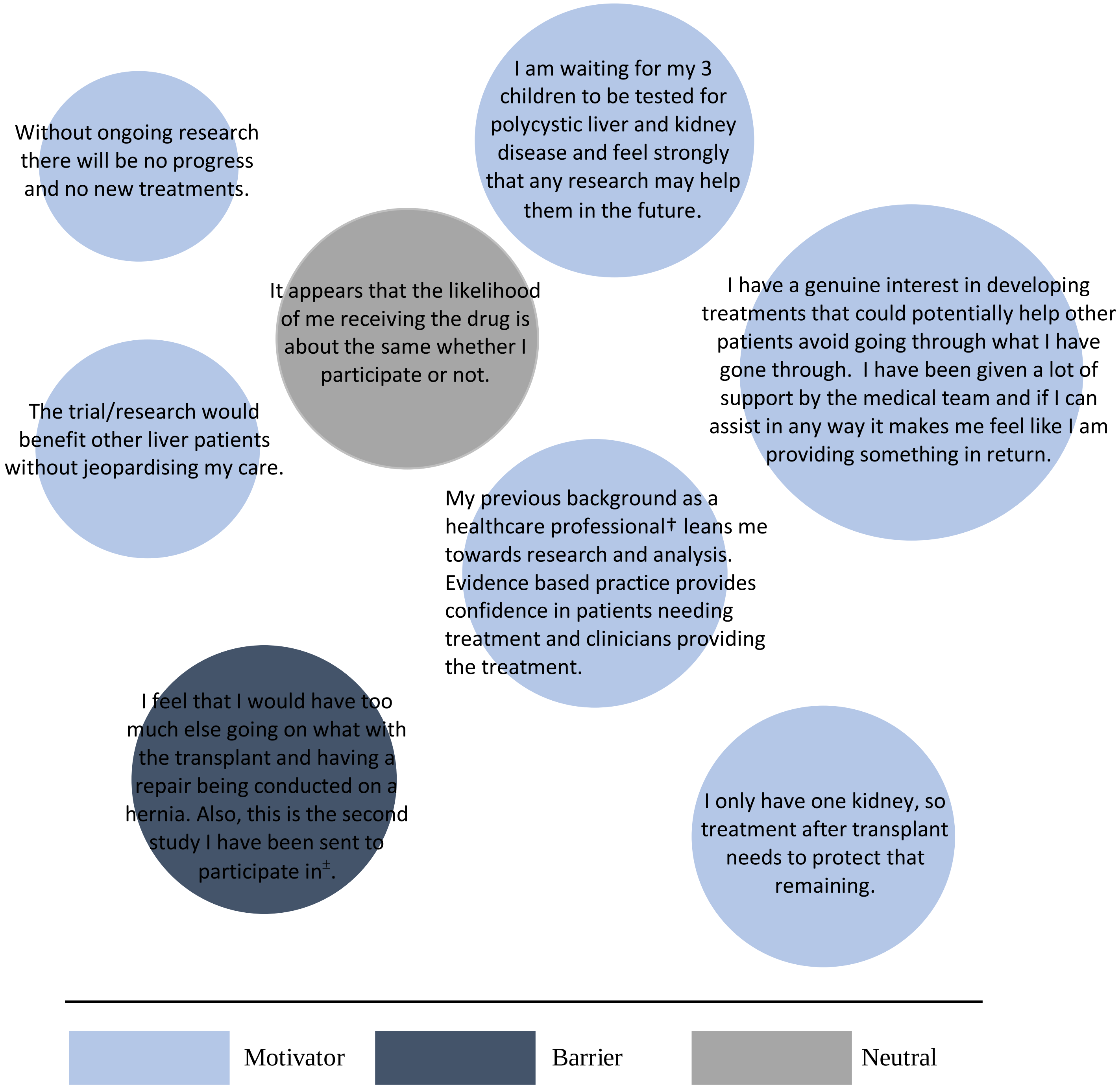

Figure 6: Motivating factors and barriers recorded by patients in response to the question “Are there any other reasons for your decision?”. 8 respondents out of 84 replied to this free-text question.

[†]response generalised from specific role for anonymity

±Comment from patient who declined to participate in RCT. All other comments are from patients who consented to participate in RCT.
