# Supplementary material for: Understanding recruitment to a randomised controlled trial (RCT) during liver transplantation: an observational mixed-methods Study Within A Trial (SWAT)
Source: BMJ Open. 2026 Jan 7;16(1):e104310. doi: 10.1136/bmjopen-2025-104310 (PMC12781991; doi:10.1136/bmjopen-2025-104310)
Supplement: Supplementary file 2 [file bmjopen-2025-104310.reviewer_comments.pdf]

PEER REVIEW HISTORY

BMJ Open publishes all reviews undertaken for accepted manuscripts. Reviewers are asked to complete a checklist review form and are provided with free text boxes to elaborate on their assessment. These free text comments are reproduced below.

ARTICLE DETAILS

Title (Provisional)

Understanding recruitment to a Randomised Controlled Trial (RCT) during liver transplantation: an observational mixed-methods Study Within A Trial (SWAT)

Authors

Coppack, Katherine Elizabeth Sarah; Kantsedikas, Ilya; Brodkin, Edgar; Loh, Ee Neng; Ambler, Gareth; Moonesinghe, S. Ramani; Fabes, Jeremy; Hannon, Vivienne; Spiro, Michael; Wagstaff, Duncan

---

VERSION 1 - REVIEW

**Reviewer**	**1**
**Name**	**Kuemmerli , Christoph**
**Affiliation**	**Clarunis University Center for Gastrointestinal and Liver Diseases, Department of Surgery**
**Date**	**01-Jul-2025**
**COI**	**None**

An interesting topic that is clearly underrepresented in clinical research.

Please also add the sometimes emergency setting and after-hours admission of transplant patients as potential barriers to recruitment.

You mention a 44% failure rate in recruiting the planned sample size. The reference is a work about trials funded by the NIHR Health Technology Assessment Programme. As University College London funds the current trial, should we expect a different failure rate?

As the recruitment process was continuously improved, e.g., by the feedback of the SWAT data analyses, there might be some outcome trend in the participants' responses. Was this assessed? One might assume that the scores were less different in the later part of the SWAT.

30 patients were included in the RCT; the number of respondents is much higher. What's the reason for the discrepancy?

Excellent tables and figures that illustrate the content nicely.

**Reviewer**	**2**
**Name**	**Wong, Tiffany Cho-Lam**
**Affiliation**	**The University of Hong Kong**
**Date**	**05-Jul-2025**
**COI**	**None**

This study is the first to embed a qualitative Study Within A Trial (SWAT) within a perioperative RCT in liver transplantation, specifically exploring patient motivators and barriers to recruitment. Recruitment to RCTs in transplantation is a well-recognized challenge, with high rates of under-recruitment threatening trial validity. This study directly addresses this issue in a complex, high-need patient group.

Could the authors provide more details on non identifiable demographic data? (e.g., age range, gender, diagnosis) were collected and, if not, discuss the implications for interpreting the findings? The manuscript mentions comparable consent rates between 2 centers. Were there any notable differences in motivators or barriers between the two centres? If so, please elaborate.

Given that most respondents were those who consented to the RCT, can the authors provide more detail on the characteristics or possible reasons for non-response among those who declined or were undecided? This would help contextualize the barriers to recruitment.

Only 8 respondents provided free-text comments. Please discuss the potential limitations of this small sample for thematic analysis and how it may affect the interpretation of qualitative findings.

VERSION 1 - AUTHOR RESPONSE

Reviewer: 1
Dr. Christoph Kuemmerli , Clarunis University Center for Gastrointestinal and Liver Diseases
Comments to the Author:
An interesting topic that is clearly underrepresented in clinical research.

Please also add the sometimes emergency setting and after-hours admission of transplant patients as potential barriers to recruitment.

Added to the abstract. Lines 131-133.
Added to discussion. Paragraph 563-575 re-formatted. Lines 567-570 explicitly mention this contributing factor.

You mention a 44% failure rate in recruiting the planned sample size. The reference is a work about trials funded by the NIHR Health Technology Assessment Programme. As University College London funds the current trial, should we expect a different failure rate?

While the 44% failure rate in achieving planned sample sizes is drawn from trials funded by the NIHR Health Technology Assessment (HTA) Programme, it serves as a valuable **benchmark/cautionary reference point.**

In this study, the associated feasibility randomised controlled trial (RCT) was **funded by an NIHR Research Award** and **sponsored by the UCL Joint Research Office**, which was responsible for study setup, conduct, and adherence to research governance standards.

Unlike many larger HTA trials, this RCT was **dual-centred,** conducted within a **defined clinical population**, and employed **focused recruitment strategies.** As such, it may have faced fewer **structural or trial design-related barriers** to recruitment that are commonly encountered in more complex or multicentre studies.

Nonetheless, the study primarily explored **patient-related recruitment challenges.** These findings may therefore complement existing data by highlighting participant-level barriers even in trials with optimised organisational frameworks.

As the recruitment process was continuously improved, e.g., by the feedback of the SWAT data analyses, there might be some outcome trend in the participants' responses. Was this assessed? One might assume that the scores were less different in the later part of the SWAT.

This was not specifically assessed, as the majority of questionnaires were distributed early in the RCT recruitment window. Additionally, as the responses were returned anonymously, it was not possible to determine the timing of individual submissions. As a result, we were unable to explore any temporal trends or changes in participant responses over the course of the study.

30 patients were included in the RCT; the number of respondents is much higher. What's the reason for the discrepancy?

The RCT had a fixed recruitment target of 30 participants, based on thresholds from a previous trial, and this was met between May 2022 and April 2023. In contrast, the qualitative SWAT component had no predetermined sample size. During the same recruitment period, questionnaires were distributed to all patients awaiting liver transplantation, regardless of whether they were enrolled in the RCT. Some respondents did not participate in the RCT—for example, because they declined consent or did not undergo a transplant—but still completed the survey. As a result, the number of SWAT respondents exceeded the number of RCT participants

Excellent tables and figures that illustrate the content nicely.

<u>Reviewer: 2</u>

Dr. Tiffany Cho-Lam Wong, The University of Hong Kong
Comments to the Author:
This study is the first to embed a qualitative Study Within A Trial (SWAT) within a perioperative RCT in liver transplantation, specifically exploring patient motivators and barriers to recruitment. Recruitment to RCTs in transplantation is a well-recognized challenge, with high rates of under-recruitment threatening trial validity. This study directly addresses this issue in a complex, high-need patient group.

Could the authors provide more details on non-identifiable demographic data? (e.g., age range, gender, diagnosis) were collected and, if not, discuss the implications for interpreting the findings? The manuscript mentions comparable consent rates between 2 centers. Were there any notable differences in motivators or barriers between the two centres? If so, please elaborate.

The questionnaires were returned anonymously, and as such, **we did not collect non-identifiable demographic data** such as age, gender, or diagnosis. We acknowledge this as a **limitation,** as it restricts our ability to contextualise responses or assess variability across patient subgroups. This point has been added to the "Limitations" section of the discussion and may be an area for further exploration in future qualitative research. Lines 596-599.

Regarding site-level differences (Table 1 supplementary files), we **did not specifically explore the differences in motivators or barriers between the two centres**. While overall consent rates were comparable, a more in-depth exploration of centre-specific factors could provide valuable insights if we were to have demographic data.

Given that most respondents were those who consented to the RCT, can the authors provide more detail on the characteristics or possible reasons for non-response among those who declined or were undecided? This would help contextualize the barriers to recruitment.

We acknowledge in the manuscript that a limitation of the study is the response bias, with the majority of completed questionnaires coming from participants who consented to take part in the RCT. As such, perspectives from those who declined or were undecided are likely underrepresented. We have now expanded the "Limitations" section and have included hypotheses about why individuals who did not wish to participate in the RCT may also have chosen not to respond to the questionnaire. Lines 601-606.

Only 8 respondents provided free-text comments. Please discuss the potential limitations of this small sample for thematic analysis and how it may affect the interpretation of qualitative findings.

We recognise that the small number of free-text responses (n=8) presents a **limitation for thematic analysis**, as it restricts the **depth and breadth** of qualitative insight and may not capture the full range of participant views. While the comments provided valuable illustrative perspectives, the findings should be interpreted with caution and regarded as **exploratory** rather than representative. This limitation has now been noted in the **discussion,** and future work could incorporate methods to encourage more in-depth qualitative feedback, such as interviews or focus groups, to strengthen thematic exploration. Lines 611-621.

Reviewer: 1 competing interests.: Not applicable

Reviewer: 2 competing interests.: NOT APPLICABLE
